# Explosive Weapons Trauma Care Collective (EXTRACCT) Clinical Practice Guideline: Transfer of Critically Injured Casualties

**DOI:** 10.1002/wjs.70456

**Published:** 2026-07-09

**Authors:** Emma Butterfield, Gavin Wooldridge, Solomon Abraha Asfaw, Mervat Alhilal, Mardi Steere, Benjamin W. Wachira, Paul Reavley, Roman Shtybel, Marius Rehn, EXTRACCT Clinical Practice Guidelines collaboration

**Affiliations:** ^1^ Intensive Care, Barts Health NHS Foundation Trust London UK; ^2^ Paediatric Intensive Care and Retrieval North West and North Wales Paediatric Transport and Retrieval Service Manchester UK; ^3^ Humanity & Inclusion Mekelle Ethiopia; ^4^ HALO Trust Amman Jordan; ^5^ Royal Flying Doctor Service SA/NT Adelaide Australia; ^6^ AMREF Flying Doctors Nairobi Kenya; ^7^ Emergency Medicine The Aga Khan University Nairobi Kenya; ^8^ Pediatric Emergency Medicine Bristol Royal Hospital for Children Bristol UK; ^9^ Medical Service of the 12th Special Forces Brigade Donetsk Oblast Ukraine; ^10^ Air Ambulance Department, Division of Prehospital Services Oslo University Hospital Oslo Norway; ^11^ Institute of Clinical Medicine University of Oslo Oslo Norway; ^12^ Department of Research and Development Norwegian Air Ambulance Foundation Oslo Norway

**Keywords:** critical care, resource limited setting, transfer, transport, trauma

## Abstract

**Introduction:**

Blast‐injured patients often present to healthcare facilities which cannot definitively meet their clinical needs. This clinical practice guideline (CPG) reviews and describes best practice for inter‐facility transfer of casualties in resource limited and conflict‐afflicted contexts where there may not be a formal referral pathway or specialized inter‐facility transfer service.

**Methods:**

A narrative literature review and synthesis of expert opinion were used to develop this CPG.

**Results:**

The guideline describes current standards and best practice in inter‐facility transfer in a low resource context—particularly during conflict when referral and transfer pathways may be overwhelmed—and advises on the selection and preparation of patients, as well as equipment and staff.

**Conclusion:**

This CPG outlines how clinicians can prepare to deliver the safest possible transfer in their context using a series of simple checklists and a flowchart to guide decision making. The evidence base for the CPG is limited by the lack of data on inter‐facility transfers in resource‐limited contexts and the difficulty in translating guidance from the global north into resource limited and conflict‐afflicted contexts.

## Graphical abstract

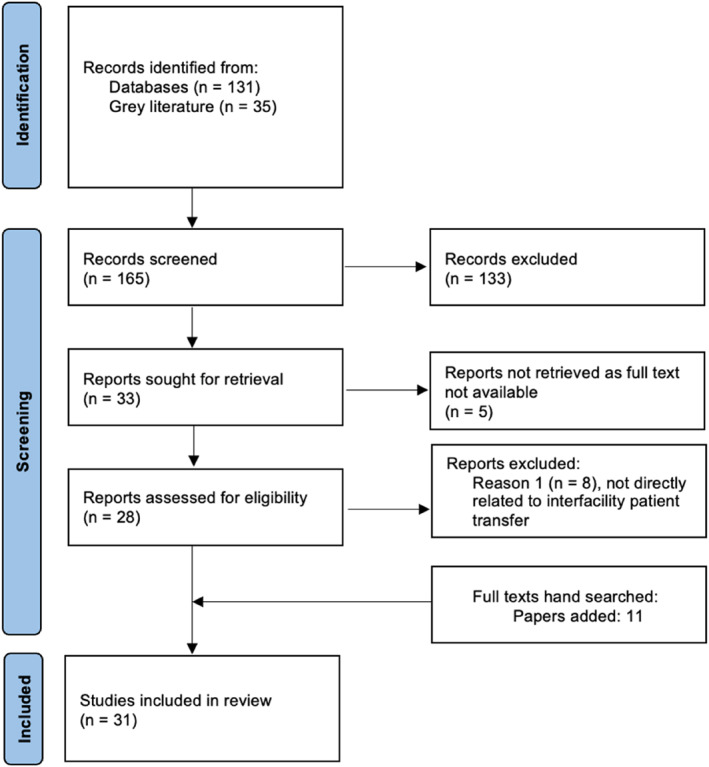

## 1 Objective

The Transfer of Critically Injured Casualties CPG provides a structured approach to the prioritization and preparation of patients with explosive and other conflict‐related injury for transfer between health facilities in resource‐limited and conflict‐afflicted health systems. Recognizing the extremely limited provision of pre‐hospital care in resource limited environments, it provides a decision support tool for clinicians and healthcare facilities to anticipate, prepare and patient transfers.

Prior guidance on combat casualty care written for military medical services presupposes access to an advanced casualty evacuation chain from point of injury to definitive care usually out of the region of conflict [1]. Most local casualties injured in conflict, and local defense forces, will have variable access to this system and will predominantly be cared for within local health systems or at nongovernmental organization (NGO) facilities with different resource constraints. This requirement will be driven by clinical and operational needs. This CPG is written to guide clinicians working within resource‐limited systems who are responsible for the triage and transfer of critically injured casualties. In modern armed conflict, most civilian injuries are caused by explosive weapons [2] and 94% of those killed by explosive weapons in populated areas (EWIPA) are civilians [3]. To reduce preventable death and disability from explosive injuries patients must be expediently transferred to facilities with surgical capabilities. This CPG outlines how safe and timely transfer can be achieved in a resource limited healthcare system.

## 2 Background

Blast‐injured patients frequently present to healthcare facilities, often run by foreign actors, whether humanitarian or military, which cannot meet their clinical needs [4]. Inter‐facility transfers comprise over 50% of ambulance journeys in some centers [5, 6], with the majority being surgical or obstetric patients [7]. Transportation of injured patients is therefore an essential, but often neglected, component of emergency care pathways [8]. Transfer must be efficient: Delays to surgical care for injured patients are associated with increased mortality [9, 10, 11, 12]. Extremity and head injuries, respectively 50% and 26% of all cases, are the most common civilian injuries [13], with compressible (43%) and non‐compressible (22%) hemorrhage leading causes of death following blast injury [14]. Blast‐injured patients must be transferred to surgical centers, even when formal interhospital transfer is not available. Triage is critical: delays in reaching surgical control are lethal to patients with non‐compressible hemorrhage but may not impact mortality in those with isolated traumatic amputations [15].

Blast injury is the predominant mechanism of civilian war trauma [2]. In addition, in a post conflict setting, civilians are frequently exposed to unexploded ordnance [16]. Blast injuries are complex and multisystemic [2, 15, 17, 18], and often not apparent on initial clinical examination [19, 20]. Healthcare facilities must anticipate a surge in patients following an attack with explosive weapons. To maximize the number of blast injured patients transferred to lifesaving surgical care, it is essential to undertake triage and risk‐stratification for transfer whilst incorporating the precise mechanism of injury. This CPG sets out a process for clinicians confronted with this horrific reality.

The World Health Organization (WHO) Emergency Care Framework outlines the components of essential emergency care [21]. It recognizes that prehospital transport and inter‐facility transport are key components of the emergency care system and has published guidance on the evaluation of pre‐hospital care systems and clinical protocols [22, 23]. However, in many places inter‐facility referral and transport pathways are absent, poorly established, or vulnerable to disruption during conflict [24, 25, 26]. The increasing use of drones also compromises evacuation and logistics including medical supply chains and prehospital transport of patients [27]. Transport times are often lengthy [28, 29], prolonged by factors including remote terrain, weather [30], and security constraints [5, 30, 31, 32]. Many patients live far from health facilities [33, 34] and rely on transportation by private vehicles to hospital, or between facilities [34, 35, 36, 37, 38, 39]. Transport vehicles may have insufficient resources [36, 39, 40, 41] or lack appropriately trained staff [41, 42] to continue care *en route* or may travel to an unsuitable facility [43], potentially contributing to excess mortality [38, 41, 44].

Globally, inter‐facility transfers are associated with adverse events in up to 68% of cases, with serious adverse events occurring in 4.2%–8.9% of transfers [45, 46]. The rate of incidents and adverse outcomes is lower in systems which use standardized protocols and checklists and have access to adequately trained staff [47, 48, 49] and equipment. In well‐resourced healthcare systems, care provided during transport should match the quality of care at the referral point [50]. Although this is often set as the standard, it may be more aspirational in certain situations. For patients with blast injuries, clinicians need to weigh the risks of delaying surgical treatment against the potential dangers associated with transferring the patient.

Scant information exists on how to undertake patient transfer in resource limited contexts. Existing guidelines predominantly describe the inter‐facility transport in high income countries [45, 47, 50, 51, 52, 53], with additional guidance on the special categories of transfer including pediatric, neonatal, and extracorporeal membrane oxygenation (ECMO). These options are not available in most resource limited contexts–and each context poses unique challenges and threats, and benefits from different skills and resources. Guidance written for high‐resource systems does not offer pragmatic solutions or risk mitigation strategies for clinicians working in resource‐limited environments. Neither does it address the ethical challenges of interfacility transfer where a patient is moved from a higher to lower capability facility for ongoing care for example from a foreign military to local national facility. This CPG therefore sets out a clinical decision tool and logical approach to support clinicians responsible for the ground transport of patients in resource limited settings. Given limited applicability in the target settings, aeromedical transport is outside the scope of this CPG. It recognizes that in a context where ambulance services are not standardized and/or staff are scarce, it may not be possible to provide the same level of care during the transfer as at the sending facility. Nonetheless, prior planning and thorough preparation of the available equipment and staff will minimize the risk of avoidable harm. Although blast‐injured patients require special consideration given the severity and complexity of their injuries, there are many commonalities to safe transport for conflict‐related injury in general. Conflict‐related injury and blast‐specific considerations are considered where relevant in the CPG.

## 3 Review Method

**FIGURE 1:**
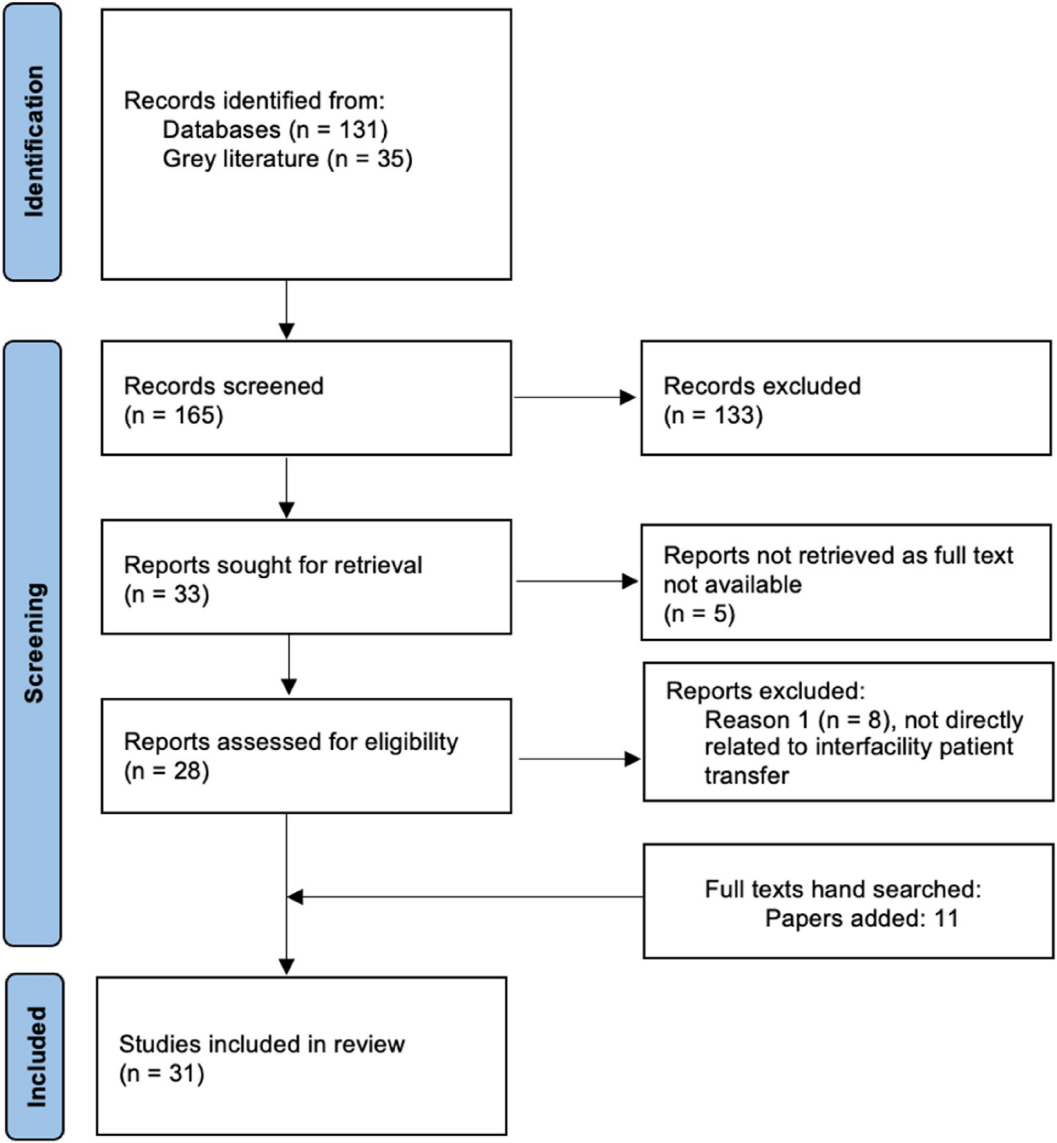
PRISMA flow diagram.

A literature search was conducted on 22/9/25 of the MEDLINE database through the PubMed portal (https://pubmed.ncbi.nlm.nih.gov/) using the MeSH Terms “Transportation of Patients”; “Developing Countries”; and “Resource‐Limited Settings”. 131 relevant articles were retrieved, none of which dealt specifically or in passing with the transport of combat injury or blast injury. Following abstract review, 33 were included: full text was available for 28 (Figure 1). 11 further papers were obtained following review of the references of included papers (Supporting Information S1: Supplementary material 1). A full systematic or scoping review was beyond the remit of this CPG.

Gray literature was searched using Google and Google Scholar with the search string “interfacility transfer” OR “patient transport” OR “interhospital transfer” AND “guideline” yielding 34 relevant guidelines, all from high‐income countries, in the first 50 results (Supporting Information S1: Supplementary material 2). Additional relevant resources were requested from sector experts resulting in the inclusion of one patient transport guideline from Ethiopia [54]. The scope of analysis was not limited to any specific distance or means of transport.

The CPG has been structured in accordance with the EXTRACCT CPG process [55] (Supporting Information S1: supplementary 3). Drawing upon their experience of transfer medicine in a variety of contexts, including resource limited and conflict areas (Supporting Information S1: supplementary material 4), the authors have translated the principles of existing guidance to resource‐limited healthcare systems. Discordant opinions were resolved by discussion until consensus was reached. Because resources and circumstances differ, these recommendations are designed as tools to help clinicians make local decisions, using the guiding consensus principles. The process followed the AGREE2 concept for developing clinical guidelines within the limitations of a small literature base [56].

## 4 Patient Transfer

Patients may be transferred between healthcare facilities for several reasons:

- Clinical: The patient requires care which is not routinely available at the sending facility (e.g., surgical or subspecialty surgical care, blood transfusion, and advanced imaging). Because of the potential for occult internal injury that may be life‐threatening even in the absence of extensive visible external trauma, most patients with injuries due to explosive weapons are likely to need imaging and/or surgical review.
- Capacity: The sending facility is temporarily unable to deliver the care the patient requires (e.g., overwhelming number of patients and damage to infrastructure or patient safety). Air‐dropped weapons are now the predominant cause of civilian combat trauma [57]—their indiscriminate nature and massive explosive impact result in many simultaneous casualties, likely to overwhelm the nearest healthcare facility.
- Consumer: The patient or their legal decision‐maker wishes for the patient to be treated in a different facility (e.g., to be closer to home/family or to minimize costs).
- Operational: Military facilities may move civilian patients according to their operational needs i.e., maintaining capacity for injured combatants to support operational effectiveness.
- Eligibility: Civilians may receive initial damage control surgery but are not eligible for ongoing care and therefore require transfer to local national or NGO facilities.

The urgency of the transfer depends on the urgency of clinical need and the local ability to meet those needs. It can be classified as emergent, urgent, or routine. Therefore, before any transfer, all injured patients must be appropriately triaged, clinically assessed, and any essential immediate treatment provided. The triage and clinical assessment of blast‐injured patients is particularly challenging as they are most likely to present in the context of a mass casualty event, and are likely to have multisystem injury [58], which clinical examination is unlikely to detect [19]. Additional challenges include the evolution of primary injury over time and the presence of significant occult injury.

**FIGURE 2:**
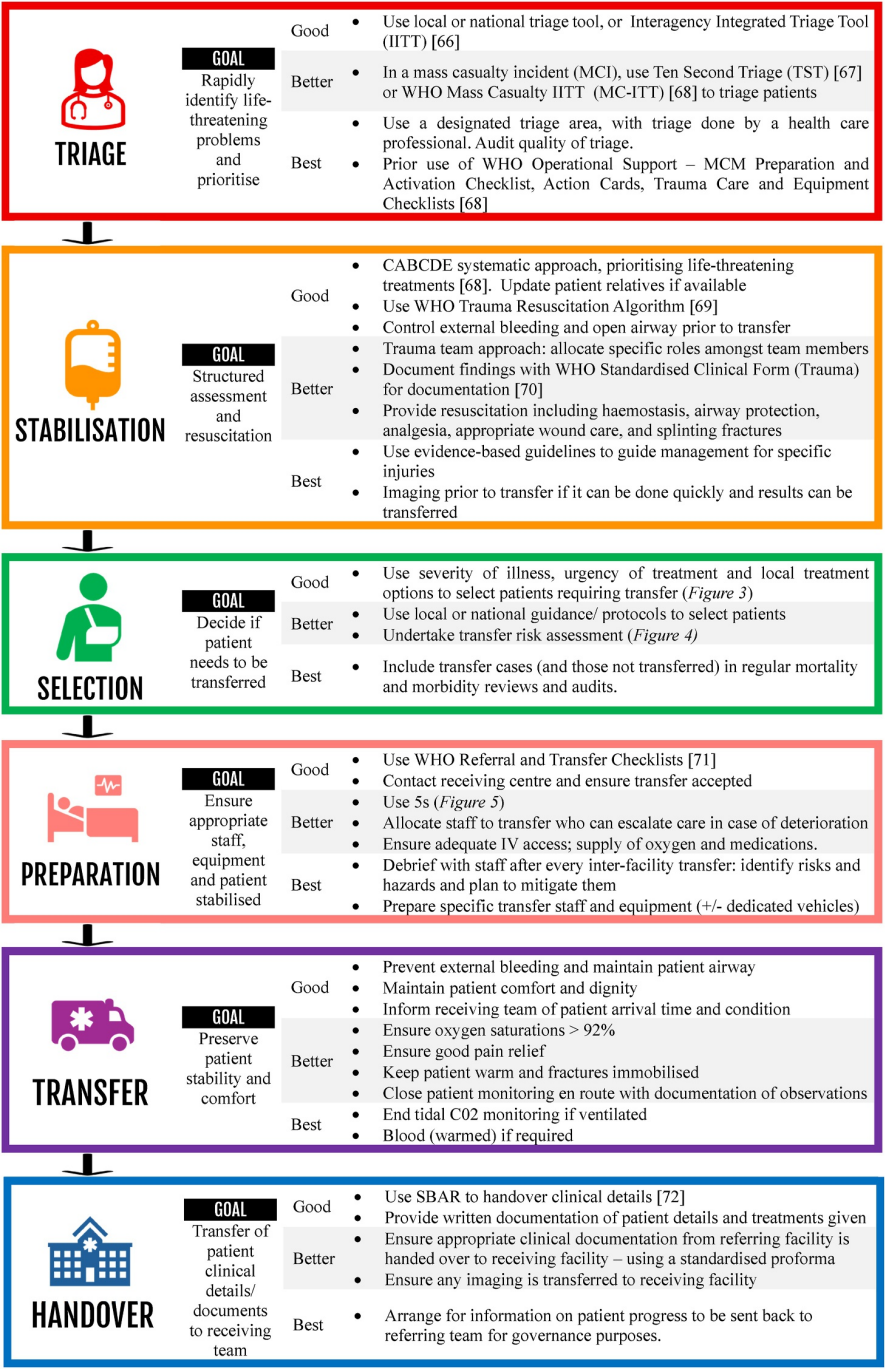
Patient transfer and tiered care recommendations. IITT, interagency integrated triage tool; IV, intravenous; MCI, mass casualty incident; MC‐ITT, mass casualty IITT; MCM, mass casualty management; SBAR, situation, background, assessment and recommendation handover tool; TST, ten second triage; WHO, World Health Organization.

Figure 2 demonstrates how safe patient transfer follows a systematic process:

1. Triage
2. Assessment and stabilization
3. Selection for transfer
4. Preparation for transfer
5. Transfer and care en route
6. Handover at receiving center or vehicle to vehicle exchange point (ramp to ramp)

The World Health Organization has published guidance on safe interfacility transfer by specialist “MEDEVAC” teams—which are well‐resourced teams dedicated to interfacility transfer [59]. This sets a standard for clinical care and governance in transfer and is an invaluable reference for any organization responsible for organizing transfers. Similarly, essential emergency and critical care (EECC) is a set of evidence‐based minimum standards for global critical care [60]. These provide a reference framework for healthcare organizations, allowing them to audit their capabilities and identify areas for improvement.

Inter‐facility transfer is not included in EECC, but its standards (Box 1) can be adapted to define the standard of care which each type of healthcare facility should provide when transferring patients (Figure 2). Healthcare managers, administrators, and lead clinicians should ensure their healthcare facility can provide a level of care during transfer commensurate with its WHO level.

**BOX 1 Reference standards for essential emergency and critical care [60, 61, 62].**

1

**Table:** 

Level of care	Definition	Target facility
Good	Essential critical care achievable with minimal equipment and staff. Should be available to all critically ill patients [60]	WHO level 1 facilities (small hospitals/health centres with sparsely equipped procedure or operating room)
Better	Next level of critical care, requiring additional resources and clinical expertise (includes all good level of care)	WHO level 2 facilities (district/provincial hospitals with adequately equipped major and minor operating rooms)
Best	Advanced level of critical care, with access to intensive care resources and staff (includes all better level of care)	WHO level 3 facilities (referral hospitals with adequately equipped operating rooms and intensive care facility)

This CPG provides guidance, aligned with these international standards, for clinicians who need to provide interfacility transfer without the support of a well‐established and governed formal transfer service. The provision of “good” care in patient transfer does not require any specific equipment: adequate training in triage, systematic patient assessment, first aid skills, referral, and handover will yield the greatest improvements in care at this level. This is true even in blast‐injured patients, in whom basic control of external hemorrhage [14] and ensuring airway patency may be lifesaving. The recommended levels of monitoring, equipment and medication available in healthcare facilities of each level have been described previously [61, 62].

## 5 Triage

All injured patients should be triaged using a validated triage tool which identifies and prioritizes patients with life‐threatening problems. The interagency integrated triage tool is validated for acuity‐based triage by clinical staff of adults and children in resource limited environments [63]. If healthcare facilities are overwhelmed, a tool designed for non‐clinical first responders, such as the ten second triage [64], can be used to prioritize casualties for emergency treatment, followed by formal triage. The WHO mass casualty interagency integrated triage tool is augmented by preparation and equipment checklists and action cards [65]. A “mass casualty” event is defined by the facility being overwhelmed by the number or type of patients [65]—in small facilities with few staff, a small number of seriously injured patients may overwhelm local capabilities. Mass casualty events are inevitable when explosive weapons are deployed in populated areas: staff in all facilities should plan for these events and consider how they will triage and transfer patients in such an eventuality.

## 6 Assessment and Stabilization

Following triage, the clinical assessment of injured patients should use a structured approach, such as the WHO trauma resuscitation algorithm in the basic emergency care (BEC) guidance [66], or tactical combat casualty care [1]. Resuscitation of the patient occurs simultaneously with patient assessment. The clinical assessment must be thorough and well‐documented as it is used to determine the patient's clinical needs, including the need for transfer. The clinical assessment must prioritize an understanding of the exact mechanism of injury, which defines the likely injury pattern: distance from the blast epicenter, type of device, use of personal protection equipment, and protection from the blast wave and debris are key determinants of the probability of injury in civilian blast injury [67]. Trauma proformas and toolkits, such as that produced by the WHO [68] and EXTRACCT [69], facilitate rapid reproducible standardized documentation of injuries, and have been successfully implemented in resource limited settings [70].

## 7 Selection for Transfer

There is little literature on patient selection for transfer. If the patient's clinical needs cannot be well‐met locally, the potential benefit of the transfer must be weighed against the risks of transfer. This is a case‐by‐case and individualized decision, based on an assessment of the patient, their likely injuries, and care available locally and in a receiving facility. Every patient and context are unique, and the risks and benefits of a transfer will vary accordingly (Box 2): clinicians at all levels must be prepared to make thoughtful and principled decisions.

**BOX 2 Case examples.**

1

**Table:** 

Example case 1
A 24‐year‐old woman is carried into a local health care facility by her husband. They were pedestrians injured when a nearby house was bombed. The blast wave propelled them into a wall and struck by debris from the blast. She is bleeding profusely from a partially amputated lower leg and is unconscious. Her husband is covered in dust with a deformed right wrist.
The staff triage the patients and recognize that the woman has life threatening injuries which requires urgent treatment. They apply pressure to the wound with sterile gauze but fail to fully stop the hemorrhage. A tourniquet is therefore applied above the wound, which achieves good hemorrhage control. The local clinic staff suspect a concomitant head injury as she remains unconscious but have ensured airway patency and taken appropriate measures to protect her cervical spine as best as possible. The local hospital is 25 min' drive away, but it is currently not safe to transport the patient because of ongoing bombardment. In the meantime, the local staff fully examine for other injuries and maintain pressure on the bleeding area. She has a palpable radial pulse. The husband is also now attended too: his broken wrist is splinted, and he receives simple analgesia. There is no formal inter‐facility transfer network. The husband calls a family member who has a car, who offers to transport them to the hospital. The staff perform a transfer risk assessment and a pre‐transfer checklist. A nurse will accompany them. When the bombing has subsided, the wife is laid in the recovery position on the back seats of the car, secured by seatbelts, with an oropharyngeal airway in place. The nurse sits in the passenger seat from where they can monitor her airway and pulse. She is thereby transported to the local hospital.
Example case 2
A 2‐year‐old child presents to a district hospital with circumferential deep partial thickness burns to her right hand, forearm, and scalp, from an explosion. She has no other injuries, and her airway is not compromised. The team recognize the circumferential burn to the hand and limb are limb‐threatening (she may develop compartment syndrome or contractures). She is likely to need burn debridement. The treatment is urgent: Early debridement is more effective, and outcomes in burn care are better in expert centers. Local treatment (burn cooling; patient warming; and analgesia) is effective first, but is not the gold standard, and the local team are not competent to perform fasciotomies or surgical debridement. The team provide analgesia, burn cooling, and patient warming, and refer her for urgent transfer for a limb‐threatening injury.

**FIGURE 3:**
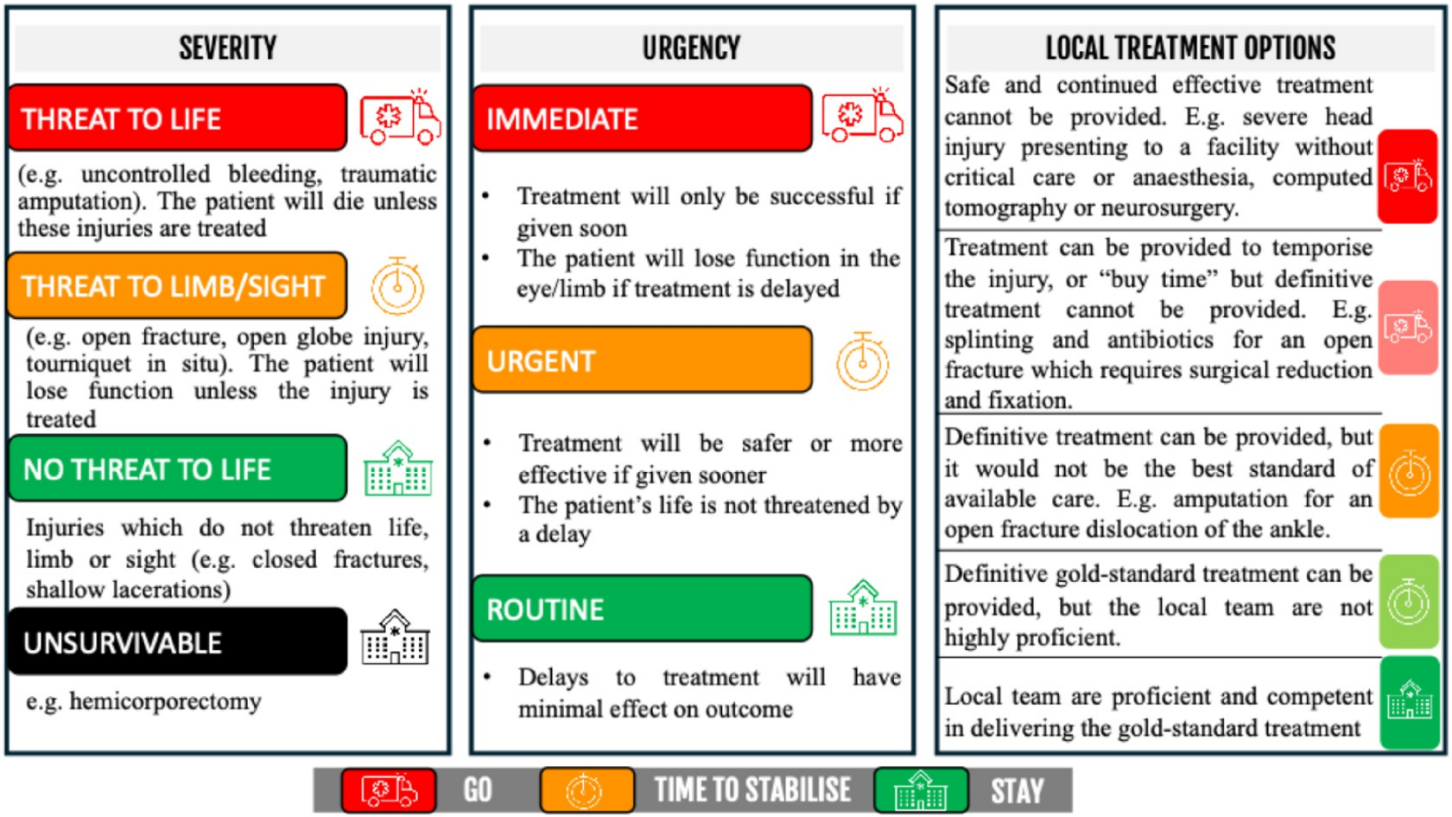
Patient needs assessment.

The decision support tool (Figure 3) provides a framework for structured decision making, using three key questions:

1. Severity: How severe is the injury?
2. Urgency: How urgent is the treatment?
3. Treatability: What care can be delivered locally?

When assessing severity of injury and urgency of treatment, clinicians should bear in mind the precise mechanism of injury, which is predictive of energy transfer and anatomical injuries, and therefore of the severity of injury, and the usual trajectory of injury progression. Blast‐injured patients have a higher mortality rate than other traumatic injury mechanisms [17] and often have complex multi‐system injuries, although the specific pattern of injury varies with the location and type of blast [17, 71]. Patients with blast‐related burns have particularly high mortality and are prone to worsening edema (which may threaten the airway) and systemic inflammatory response syndrome, including adult respiratory distress syndrome. Because of primary blast effects, patients with blast injury may develop worsening respiratory distress as blast lung syndrome develops over 12–48 h, and signs of hollow viscus rupture may take hours to evolve [72].

The most severe injury should be used to assess the patient. The transfer of patients with an injury in need of immediate treatment should not be delayed for the treatment of their less severe injuries (e.g., suturing of wounds). Patients with untreatable or unsurvivable injuries (in the context of local capability) will not benefit from transfer, but injuries which appear lethal may be survivable with advanced care. If there is any doubt about the potential survivability (e.g., severe burns) then consultation with expert clinicians at the receiving facility may be very helpful. If this is not possible, and doubt remains about the survivability of the injury, transfer to a higher level of care for expert review is recommended as the least restrictive option.

The decision to transfer a patient depends upon where their needs are best met. This is determined by the relative capabilities of the sending and receiving facility and requires close liaison with other healthcare providers. Where there is uncertainty, senior clinical decision makers, and people with knowledge of local transport options and clinical facilities, should be consulted. Every effort should be made to avoid initiating treatments which will delay or complicate necessary transfers.

## 8 Preparation for Transfer

Proper preparation reduces the risks inherent in removing a patient from the healthcare environment and placing them in the transfer environment with limited equipment and staff. It also considers the risks of the journey, such as road traffic accidents, delays, and exposure to conflict, heat, and cold. Detailed advice on navigating checkpoints, curfews, drone avoidance, and conflict zones is location‐specific and beyond the remit of the guideline—clinicians should utilize local knowledge, official guidelines, and their community connections.

**FIGURE 4:**
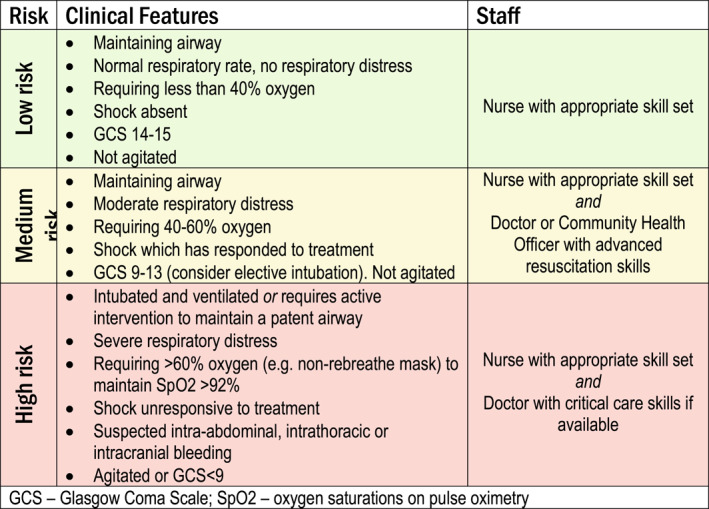
Modified ICS risk assessment tool [47]. Patients are only classed as low risk if they have no medium risk features. If a patient has any high‐risk features, they are a high‐risk patient.

Where there are no formal inter‐facility transfer services, those patients that have access to private transport may be able to arrange their own transfer. Patient autonomy must be respected, but it remains the responsibility of the treating clinicians to advise the patient and/or their representative of the risks and benefits of the transfer, and to ensure that it is conducted as safely as possible (e.g., by providing staffing where appropriate). In a mass casualty situation, ambulance services can be quickly overwhelmed, requiring the use of local private vehicles, transfer without accompanying staff or the transfer of more than one patient per vehicle. To the authors' knowledge, no peer‐reviewed literature or guidelines exist on how clinicians should assess or mitigate the risks of this. In this circumstance, as ever, the clinical risks of a transfer should be thoughtfully assessed by a senior clinician. Tools have been developed to guide such processes [47, 73] and can be adapted to the local context (Figure 4). If there is a risk of the patient deteriorating and needing clinical intervention en route, appropriately skilled staff should accompany them if such staff are available without compromising the ability to care for other casualties.

### 8.1 Challenging Decision Making

Where the need for transfer outstrips a facility's capacity to staff and equip transfers, there will be difficult decisions about resource allocation. Triage tools have historically been based on utilitarian principles (to do the greatest good for the greatest number [74]) and the basic humanitarian principles of impartiality, neutrality, humanity, and independence [75]. However, different ethical principles (e.g., libertarian, egalitarian, and restorative codes) may guide local resource allocation [76] care should be distributed according to the local law and codes of ethics codes guiding physician conduct [77]. These decisions are difficult: Sometimes there are only bad options. The decision support tool (Figure 3) is designed to support structured reproducible decision making when staff are faced with overwhelming and/or complex needs. Where possible, decisions should be made collaboratively with colleagues and clinicians in the receiving facility, aiming to reach a principled consensus. Sharing responsibility for these difficult choices can help ease the cognitive and emotional burden.

### 8.2 Checklists

Most adverse events during transfer are avoidable [78]. Therefore, a resolute focus on addressing treatable life‐threatening pathology before and during transfer is essential especially when many blast injured deaths result from compressible bleeding [79]. Checklists facilitate this clarity of thought under pressure including repeated re‐evaluation of casualties.

Poor documentation and communication between centers and unfamiliarity with the environment, processes, and equipment used in transfers increases the risk of an adverse event [45, 78]. The impact of errors can be reduced by checking the equipment and the patient prior to departure, as well as the use of protocols, standardized paperwork, and checklists [80, 81]. Transfer checklists are available including the *WHO Acute Referral Form*, the *WHO Acute Transfer Checklist*, and the *WHO Interfacility Acute Transfer checklist* [68]. Additional checklists can be found in the Supporting Information S1: supplementary material 5.

**FIGURE 5:**
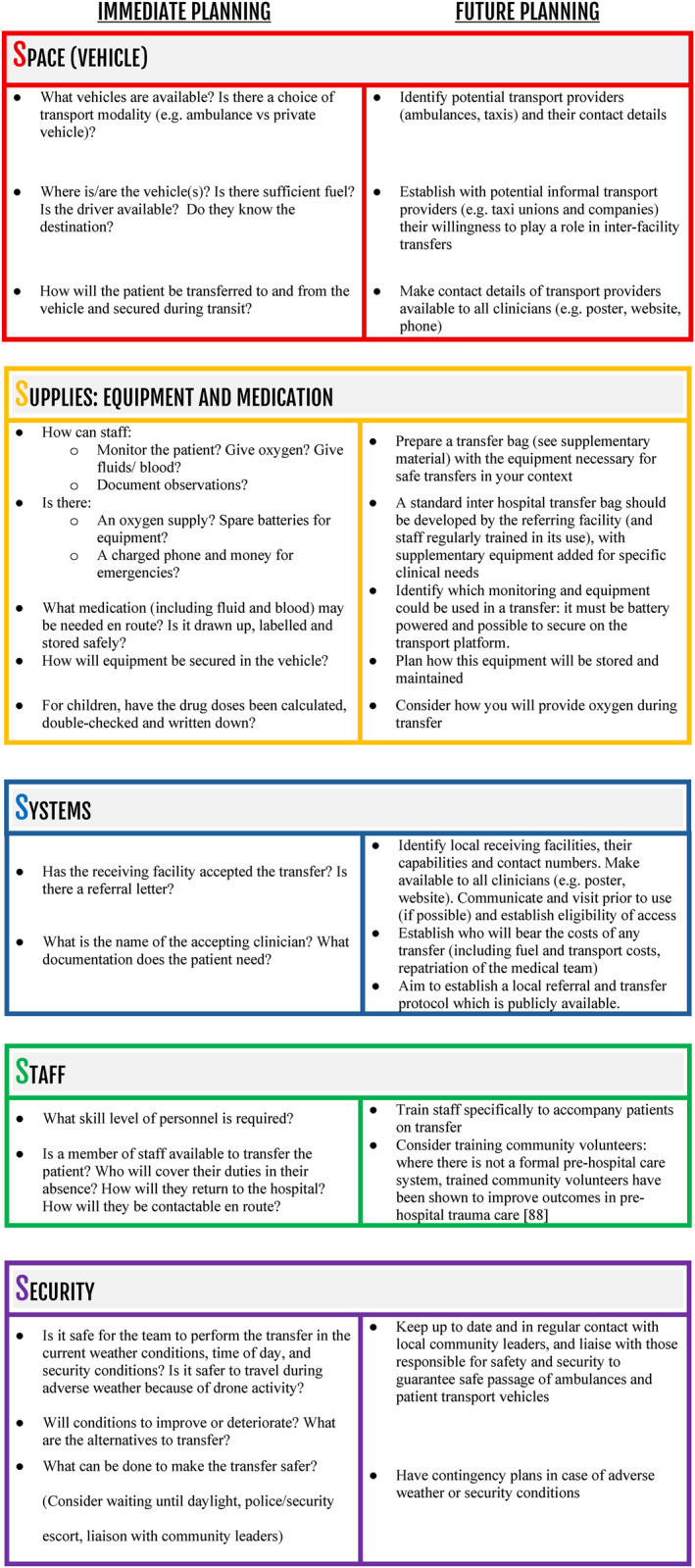
5S Immediate and future planning.

The logistical aspects of the transfer must also be considered. We have developed a cognitive aid (Figure 5), based on the 5S approach [82, 83], which can be used as a pre‐transfer logistics checklist in conjunction with a clinical transfer checklist. Delays in surgical intervention for injured patients can be life‐threatening [9, 10, 11, 12], especially in cases of blast injuries [15]. After achieving hemorrhage control and securing the airway, minimizing the time to surgical care is likely the most critical intervention for patient survival, even when non‐surgeon physicians are providing treatment [71].

## 9 Transfer and Care en Route

### 9.1 Pre‐Departure

- Discuss problems that may occur during transfer, thresholds for treatment, and what actions staff can take (e.g., a clinician might advise that if a bleeding patient no longer has a radial pulse, the transfer staff should administer a fluid bolus) [83]. Consider specific conflict injuries such as limb amputation and penetrating chest injury. Tourniquets may slip and lose control; chest seals may block. Inappropriate tourniquet use can cause harm, and it is important to know who requires one and when to consider conversion [84, 85].
- Sometimes, no effective treatment is possible in the sending facility or *en route* (e.g., patient with a threatened airway obstruction, being sent from a facility which does not have anesthetic capabilities)—in these circumstances, the transfer staff must be aware of their limitations, the need to expedite transfer, and the possibility of irreversible deterioration *en route.* These situations can be very stressful for staff particularly when the condition of the injured patient is outside their standard scope of practice, and they should be supported by their colleagues.
- Safeguarding: Ensure vulnerable children and adults have sufficient safeguards and support in place for the transfer.

During transfer (Figure 6).

- After any movement (e.g., into or out of an ambulance/private vehicle), a quick systematic cABCDE assessment [86], including vital signs, should be undertaken. It is imperative to attempt to preserve the patient's clinical status, comfort, and safety during the transfer, with priority given to:
  ◦ Ensuring external bleeding is controlled
  ◦ Ensuring airway patency, ventilation, and oxygenation
  ◦ Ensuring access to intravenous access and medications
- Good pain relief is essential: staff should provide the best‐possible analgesia.
- Vital signs. Non‐invasive blood pressure measurements are unreliable in transit [87], but pulse, respiratory rate, and conscious level should be documented regularly and contemporaneously. The health care professional responsible for the transfer should have a plan to respond to any clinical deterioration *en route.*
- In case of deterioration *en route* the transfer staff should ideally communicate with the sending clinical team, or the receiving clinical team, as they may be able to offer advice on clinical management. However, it should be assumed that communication may not be possible, so the transfer staff should be skilled and equipped to deal with any anticipated deterioration [83].
- By nature, conflict injuries are severe and evolving. Maintain access to the patient and be prepared to intervene in life‐threatening circumstances. Hemorrhage control, airway management, respiratory support, and chest decompression may be required.

FIGURE 6Conflict injury considerations during transport.

**Figure:**
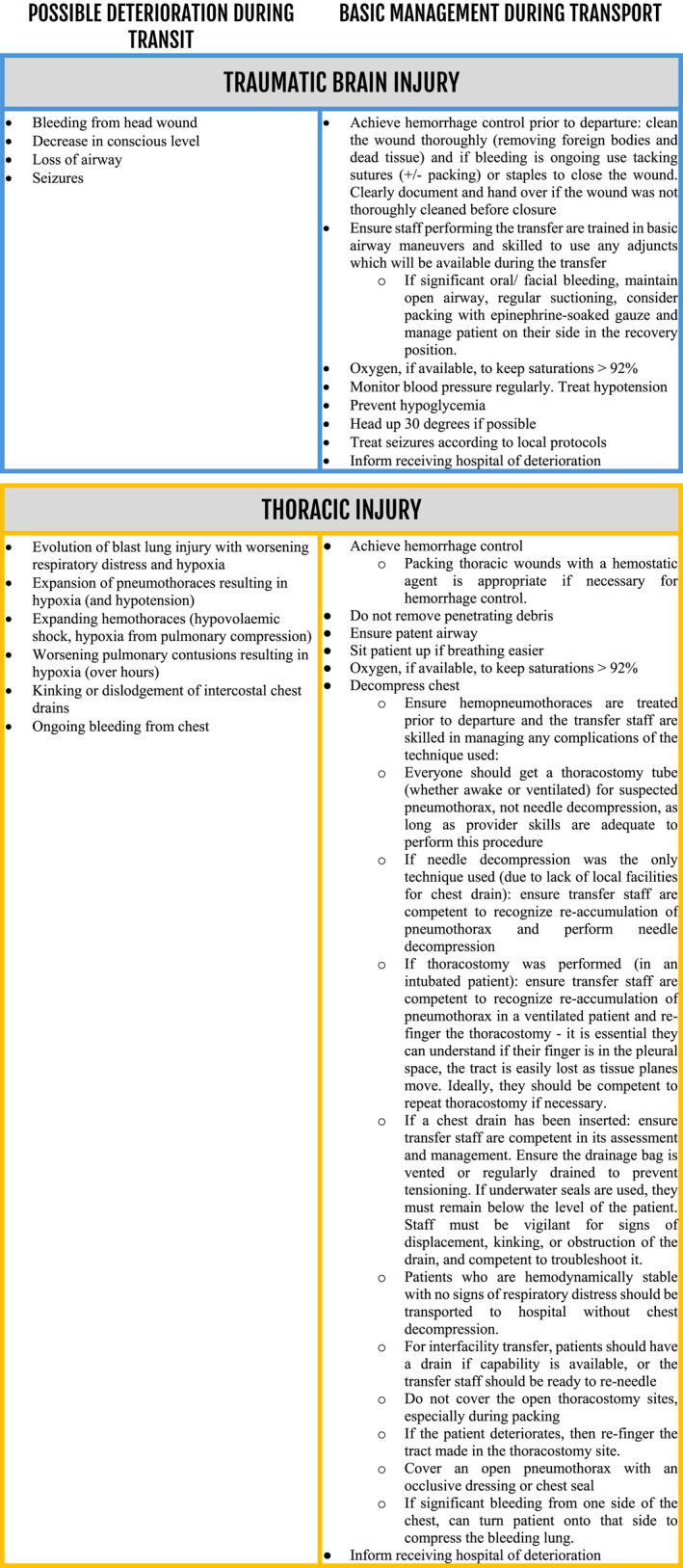

**Figure:**
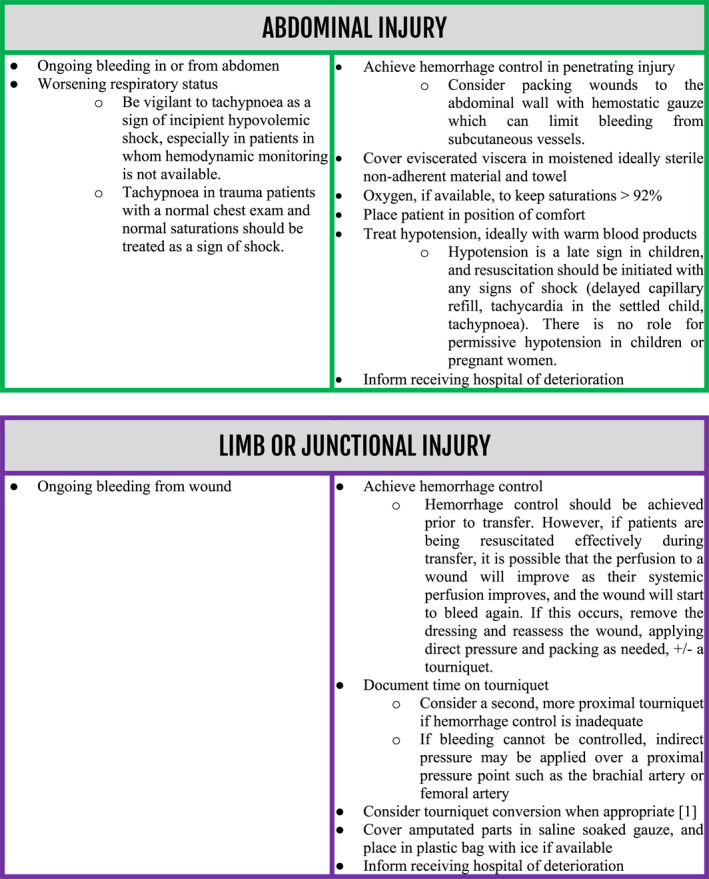

## 10 Handover at Receiving Center

Handover on arrival to the health facility should be structured using a method such the situation, background, sssessment, and recommendation (SBAR) handover tool [68].

## 11 Physical and Psychological Impact

It is important to acknowledge the physical and psychological toll of challenging transfers.

Psychological first aid strategies can be used by trained staff to identify colleagues at risk of prolonged or severe distress [88].

## 12 Performance Improvement

The risk of each individual transfer is reduced by healthcare organizations planning and preparing for patient transfers on a systems level, and by setting standards against which their performance can be audited (Figure 2).

The risks in transfer can be categorized as follows:

- Technical risks: for example lack or failure of medical equipment or inadequate communications equipment
- Non‐technical risks: for example inadequate training of staff and poor communication between staff
- Governance risks: for example lack of transfer systems and networks
- Situational risks: for example security and natural hazards.

Each healthcare system should have a transfer plan that addresses the following:

- Pre‐transport coordination and communication
- Transport personnel
- Transport equipment
- Monitoring during transport
- Documentation [52].

The transfer plan should be regularly evaluated and adapted using a quality improvement process [52].

Where there is no transfer plan, healthcare facilities and clinical leaders can adapt the 5S model to perform a practical local health systems analysis (Figure 5). Lists of basic essential equipment for transfers have been compiled and may serve as useful reference [47] but the final kit list will be highly dependent on context: staff should not take equipment they are not competent to use. In conflict contexts, facilities and clinicians must anticipate and prepare for mass casualty situations and discuss what extra resources they may be able to muster to facilitate transfers, and how they could mitigate the dangers of these informal transfers.

## 13 Performance Management and Outcome Metrics

Performance metrics must be appropriate to the specific context. Single measures are unlikely to reflect the effectiveness of the whole system, as improvements in the safety of inter‐facility transfer may shift the burden of mortality and morbidity elsewhere in the system [89]. Nonetheless, auditing performance against appropriate locally or nationally set standards, especially using standardized casualty data collection [90], can drive systems improvement.

Structured debrief of cases allows meaningful qualitative feedback and ensures lessons are learned from critical incidents, near‐misses, and best practice, and can guide changes in practice [91].

## 14 Conclusion

This CPG examines the evidence around best practice in inter‐facility transfer of injured patients in a resource limited setting, with a particular focus on local casualties of war, who are including those injured by explosive weapons. It outlines how clinicians in any context can prepare to deliver the safest‐possible transfer using a series of simple checklists and a flowchart to guide decision making.

It is not possible to make specific recommendation about staffing or equipment, due to the immense variability in contexts. Instead, the approach taken here is to provide decision support tools to help clinicians in resource‐limited contexts make structured, reproducible, and defensible decisions in very challenging and sometimes overwhelming circumstances. Clinicians in resource‐limited contexts are the experts on their environment, its limitations, and the capacity of the systems in which they work. These tools aim to empower them to leverage their expertise and skills to deliver the best possible care.

These tools are limited by their lack of alignment with local systems of care, as they are designed to be universally accessible and suitable for use in any context. They are designed to be adapted and incorporated into local systems, by the staff working there, as has previously been done for clinical guidelines on medical emergencies in primary care [92].

The evidence base for the tools, and lack of specific advice is limited by the lack of data on inter‐facility transfers in resource‐limited contexts and the difficulty in translating guidance from high‐resource settings into resource limited contexts. Future work should explore the demographics of inter‐facility transfers and utility of transfer checklists in settings other than high‐income countries. Critical analysis of these tools, by clinicians working in resource‐limited settings, will yield the greatest benefits in terms of harnessing their knowledge and experience to improve patient care globally, and following this work, this CPG should be updated to incorporate the lessons learned.

## Author Contributions

**Emma Butterfield:** conceptualization, methodology, writing – original draft, writing–review and editing, project administration. **Gavin Wooldridge:** conceptualization, methodology, writing – original draft, writing–review and editing, project administration. **Solomon Abraha Asfaw:** writing – review and editing. **Mervat Alhilal:** writing – review and editing. **Mardi Steere:** writing – review and editing. **Benjamin W. Wachira:** writing – review and editing. **Paul Reavley:** writing – review and editing. **Roman Shtybel:** writing – review and editing. **Marius Rehn:** writing – review and editing.

## Funding

The authors have nothing to report.

## Ethics Statement

The authors have nothing to report.

## Conflicts of Interest

There are no financial or ethical conflicts of interest for any authors. The lead authors (G.W. and E.B.) are members of EXTRACCT. EB sits on the medical board of HALO, a mine action charity.

## Supporting information


Supporting Information S1

## Data Availability

The authors have nothing to report.
