# Supplementary material for: Explosive Weapons Trauma Care Collective (EXTRACCT) Clinical Practice Guideline: Transfer of Critically Injured Casualties
Source: World J Surg. 2026 Jul 9;50(8):2455–70. doi: 10.1002/wjs.70456 (PMC13460947; doi:10.1002/wjs.70456)
Supplement: Supplementary file 1 [file WJS-50-2455-s001.docx]

Supplementary Material 1

*Full text articles*

|  | **Year** | **Author** | **Journal** | **PMID** | **Ref number** | **Country** | **Type of paper** | **Inter facility transfer?** | **Trauma patients?** | **Summary/ Title** | **Utilised in CPG** |
| --- | --- | --- | --- | --- | --- | --- | --- | --- | --- | --- | --- |
| 1 | 2021 | Purcell LN | World J Surg | 33164113 | 40 | Malawi | Retrospective observational | No | Yes | Police Transportation Following Vehicular Trauma and Risk of Mortality in a Resource-Limited Setting | Yes |
| 2 | 2019 | Haviland Mize C | High altitude Medicine and Biology | 31460794 |  | Bhutan | Retrospective observational | ? | Yes | Helicopter Critical Care Retrieval in a Developing Country: A Trauma Case Series from Bhutan | No |
| 3 | 2017 | Patel A | BMC Health Services Research | 29197385 |  | Brazil | Prospective observational, qualitative | No | Yes | Qualitative evaluation of trauma delays in road traffic injury patients in Maringá, Brazil | No |
| 4 | 2024 | Adem M A | BMC Emergency Medicine | 38735937 |  | Ethiopia | Prospective, observational | Yes | Yes | The practice and determinants of ambulance service utilization in pre‑hospital settings, Jimma City, Ethiopia | No |
| 5 | 2016 | Tansley G | Prehosp Emerg Care | 27074588 |  | Ghana | GIS mapping of ambulance centres | - | - | Population-level spatial access to prehospital care by the National Ambulance Service in Ghana | No |
| 6 | 2023 | Jana A | Nature Portfolio | 38040797 | 5 | India | Mixed methods, prospective, | Yes | Yes | Examining district‑level disparity and determinants of timeliness of emergency medical services in Maharashtra, India | Yes |
| 7 | 2021 | Singh J | BMJ open | 34230014 | 43 | India | Prospective observational | Yes | No | Transport characteristics and predictors of mortality among neonates referred to a tertiary care centre in North India: a prospective observational study | Yes |
| 8 | 2008 | Crandon I | Int Journal of Surgery | 18789880 | 44 | Jamaica | Prospective, descriptive | Yes | Yes | Inter-hospital transfer of trauma patients in a developing country: A prospective descriptive study | Yes |
| 9 | 2013 | Hardcastle T | World J Surgery | 23196339 | 45 | South Africa | Prospective, descriptive | No | Yes | The Prehospital Burden of Disease due to Trauma in KwaZulu-Natal: The Need for Afrocentric Trauma Systems | Yes |
| 10 | 2022 | Suzuki T | World J Surgery | 35041060 |  | Laos | Prospective observational | No | Yes | Relationship Between Prehospital Time and 24-h Mortality in Road Traffic-Injured Patients in Laos | No |
| 11 | 2013 | Wilson A | Int J Gynecology and Obstetrics | 23806250 | 31 | LMIC | SR | Yes | No | Systematic review and thematic synthesis of qualitative studies on maternal emergency transport in low- and middle-income countries | Yes |
| 12 | 2016 | Abdulraheem M | Pan African Medical Journal | 27800071 | 41 | Nigeria | Prospective, descriptive | Yes | No | Neonatal transport practices in Ibadan, Nigeria | Yes |
| 13 | 2008 | Spector JM | Trop Doctor | 18302880 |  | El Salvador | Commentary | Yes | No | Coordinated neonatal transport: advancing newborn care in resource-limited settings | No |
| 14 | 2008 | Khilnani P | Indian Journal of Pediatrics | 18759088 |  | India | Commentary | Yes | Yes | Transport of Critically ill Children: How to Utilize Resources in the Developing World | No |
| 15 | 2005 | Kobusingye O | Bulletin of the World Health Organisation | 7778210 | 8 | LMIC | Review | Yes | Yes | Emergency medical systems in low- and middle-income  countries: recommendations for action | Yes |
| 16 | 2008 | Venticinque S | Critical Care | 18594254 |  |  | Review | Yes | Yes | Critical care in the austere environment: Providing exceptional care in unusual places | No |
| 17 | 2003 | Duke T | Intensive Care Med | 14560763 |  |  | Editorial | Yes | Yes | Transport of seriously ill children: a neglected global issue | No |
| 18 | 2020 | Truche P | PLOS One | 33156837 | 7 | Columbia | Retrospective analysis | Yes | Yes | Publicly funded interfacility ambulance transfers for surgical and obstetrical conditions: A cross sectional analysis in an urban middle-income country setting | Yes |
| 19 | 2015 | Jacobs B | Int Journal of Health Planning and Management | 25677821 | 33 | Cambodia | Mixed methods, qualitative, prospective observational | Yes | Yes | Ambulance services as part of the district health system in low-income countries: a feasibility study from Cambodia | Yes |
| 20 | 2024 | Thielmann B | Internal and emergency medicine | 39120855 |  | Ukraine | Commentary | No | Yes | Field report: ambulance service in Ukraine during weaponized conflict | No |
| 21 | 2018 | Bourn S | BJA education | **33456812** |  | UK | Review | Yes | Yes | Transfer of the critically ill adult patient | No |
| 22 | 2024 | Badkur M | Journal of emergencies, trauma and shock | 39552825 |  | India | Prospective observational | Yes | Yes | Factors influencing treatment delays in trauma patients:  A three‑delay model approach | No |
| 23 | 2023 | Werner K | Bmc emergency medicine | 37005602 | 26 | Conflict | Sr | Yes | Yes | Emergency care in post‑conflict settings: a systematic literature review | Yes |
| 24 | 2015 | Suryanto M | Prehospital and disaster medicine | 27938449 |  | LMIC | Review | No | Yes | Ems systems in lower-middle income countries: a literature review | No |
| 25 | 2017 | Zakariah A | Prehospital and disaster medicine | 27938469 | 6 | Ghana | Commentary | No | No | The birth and growth of the national ambulance service in Ghana | Yes |
| 26 | 2024 | Sharma B | American journal of obstetrics and gynecology | 38431464 |  | Nepal | Commentary | Yes | No | Reducing maternal mortality in low- and middle-income countries: the Nepalese approach of helicopter retrieval | No |
| 27 | 2003 | Hatherill M | Intensive care medicine | 12897999 |  | South Africa | Prospective descriptive | Yes | Yes | Transport of critically ill children  In a resource-limited setting | No |
| 28 | 2017 | Kim J | Prehospital and disaster medicine | 28748771 |  | Liberia | Prospective, mixed methods | Yes | Yes | Acute care referral systems in Liberia: transfer  And referral capabilities in a low-income country | No |
| 29 | 1999 | Altintas K | European journal of emergency medicine | 10646926 |  | Turkey | Retrospective, descriptive analysis | Yes | Yes | Costing of an ambulance system in a developing country, turkey: costs of Ankara emergency aid and  Rescue services' (ears) ambulance system | No |
| 30 | 2020 | Ethiopian Ministry of Health | National Referral Guideline | <https://www.scribd.com/document/878921923/National-Referral-Guideline-2> |  | Ethiopia | Guideline | Yes | Yes | Ethiopian Ministry of Health: National Referral Guideline | No |
| 31 | 2025 | WHO | Medical evacuation in emergencies  A guidance for medical teams and  specialized care teams | <https://www.who.int/publications/b/71393> | 62 | WHO | Guideline | Yes | Yes | Medical evacuation in emergencies: A guidance for medical teams and specialized care teams | No |

Supplementary Material 2

*Grey literature using Google Search Top 50 Results*- “interfacility transfer" OR "patient transport" OR "interhospital transfer" AND guideline

| **WEBSITE** | **COUNTRY** | **TYPE** |
| --- | --- | --- |
| <https://epiguard.com/patient-transport-101-intrahospital-interhospital-transport/> |  | Blog |
| <https://www.intechopen.com/chapters/57973> |  | Book |
| <https://www.bluecrossnc.com/providers/policies-guidelines-codes/commercial/medical/updates/inpatient-interfacility-transfers> |  | Insurance policy |
| <https://provider.healthybluemo.com/medpolicies/healthybluemo/active/gl_pw_d089204.html> |  | Insurance policy |
| <https://provider.healthybluemo.com/medpolicies/healthybluemo/active/gl_pw_d089204.html> |  | Insurance policy |
| <https://pmc.ncbi.nlm.nih.gov/articles/PMC4966347/> |  | Literature |
| <https://pmc.ncbi.nlm.nih.gov/articles/PMC9842466/> |  | Literature |
| <https://www.bjanaesthesia.org.uk/article/S0007-0912(17)31237-0/fulltext> |  | Literature |
| <https://sjtrem.biomedcentral.com/articles/10.1186/s13049-019-0604-8> |  | Literature |
| <https://www.researchgate.net/publication/261070225_When_place_and_time_matter_How_to_conduct_safe_inter-hospital_transfer_of_patients> |  | Literature |
| <https://academic.oup.com/milmed/article/189/9-10/e2229/7633800> |  | Literature |
| <https://applications.emro.who.int/imemrf/Saudi_J_Anaesth/Saudi_J_Anaesth_2014_8_1_104_113.pdf> |  | Literature |
| <https://www.sciencedirect.com/science/article/abs/pii/S1755599X17300046> |  | Literature |
| <https://ccforum.biomedcentral.com/articles/10.1186/s13054-015-0749-4> |  | Literature |
| <https://www.researchgate.net/publication/366765063_Patient_care_during_interfacility_transport_a_narrative_review_of_managing_diverse_disease_states> |  | Literature |
| <https://www.facetsjournal.com/doi/10.1139/facets-2023-0209> |  | Literature |
| <https://primarycare.northeastlondon.icb.nhs.uk/wp-content/uploads/2024/02/LAS-inter-facility-transfer-guidance.pdf> | UK | Local |
| <https://www.southaccnetworks.nhs.uk/media/South_West_CCN/guidelines/SWCCN%2520Guidelines%2520for%2520the%2520inter-%2520and%2520intra-hospital%2520transfer%2520of%2520critically%2520ill%2520adult%2520patients%2520(February%25202020)%2520(2).pdf> | UK | Local |
| <https://www.gmccmt.org.uk/wp-content/uploads/2024/06/Transfer-of-the-Critically-Ill-Adult-GM-CCN-2024.pdf> | UK | Local |
| <https://doclibrary-rcht.cornwall.nhs.uk/DocumentsLibrary/RoyalCornwallHospitalsTrust/Clinical/CorporateClinical/SafeTransferOfPatientsBetweenCareAreasOrHospitalsPolicy.pdf> | UK | Local |
| <https://www.clinicaltoolkit.co.uk/wp-content/uploads/2019/09/SWASFT-guidance-for-healthcare-professionals-requesting-an-ambulance-for-a-patient.pdf> | UK | Local |
| <https://www.wyccn.org/uploads/6/5/1/9/65199375/final_pdf_ccstt-workbook_05-2021_v6.pdf> | UK | Local |
| <https://www.wyccn.org/transfer.html> | UK | Local |
| <https://accountf2197fd5.blob.core.windows.net/payload/documents/interfacility-management-guidelines-rev-2020.pdf> | USA | Local |
| <https://www.sahealth.sa.gov.au/wps/wcm/connect/public%2Bcontent/sa%2Bhealth%2Binternet/resources/policies/inter-facility%2Btransfer%2Bpolicy> | Australia | Local |
| <http://miemss.org/home/Portals/0/Docs/Guidelines_Protocols/Interhospital-Transfer-Manual-20211216.pdf> | USA | Local |
| <https://www.albertahealthservices.ca/ems/Page16336.aspx> | Canada | Local |
| <https://www.dhs.wisconsin.gov/ems/wi-interfacilitytransportguidelines-2006.pdf> | USA | Local |
| <https://flbog.sip.ufl.edu/wp-content/uploads/2021/05/AMC-PSO-Interhospital-Transfers.pdf> | USA | Local |
| <https://www.oregon.gov/oha/PH/PROVIDERPARTNERRESOURCES/EMS/EMSFORCHILDREN/Documents/EMSC_Pediatric_Interfacility_Transfer_Guide.pdf> | USA | Local |
| <https://www1.health.nsw.gov.au/pds/ActivePDSDocuments/PD2025_002.pdf> | Australia | Local |
| <https://www.health.qld.gov.au/system-governance/policies-standards/health-service-directives/patient-access-to-care/protocol-for-management-of-inter-hospital-transfers> | Australia | Local |
| <https://www.sahealth.sa.gov.au/wps/wcm/connect/public%2Bcontent/sa%2Bhealth%2Binternet/resources/policies/inter-facility%2Btransfer%2Bpolicy> | Australia | Local |
| <http://miemss.org/home/Portals/0/Docs/Guidelines_Protocols/Interhospital-Transfer-Manual-20211216.pdf> | USA | Local |
| <https://www.england.nhs.uk/long-read/national-framework-for-inter-facility-transfers/&ved=2ahUKEwjr3ueDsZ-QAxVBUEEAHer_A7UQFnoECAQQAQ&usg=AOvVaw3z2EwaqVihdNM0f8XnGN-W> | UK | National |
| <https://gettingitrightfirsttime.co.uk/wp-content/uploads/2025/07/Inter-facility-transfer-of-critically-ill-or-deteriorating-patients-FINAL-June-2025.pdf> | UK | National |
| <https://www.nice.org.uk/guidance/ng94/evidence/34.standardised-systems-of-care-for-intra-and-interhospital-transfers-pdf-172397464673> | UK | National |
| <https://www.ficm.ac.uk/sites/ficm/files/documents/2021-10/Transfer_of_Critically_Ill_Adult.pdf> | UK | National |
| <https://www.england.nhs.uk/publication/inter-facility-transfers-framework/&ved=2ahUKEwjr3ueDsZ-QAxVBUEEAHer_A7UQFnoECCAQAQ&usg=AOvVaw11UPqrrp0SYnGiHvCRvg5w> | UK | National |
| <https://rightdecisions.scot.nhs.uk/media/1717/05-ift-booking-guide-nov-2020.pdf> | UK | National |
| <https://www.ems.gov/assets/Interfacility_Transfers.pdf> | USA | National |
| <https://www.nice.org.uk/guidance/ng94/documents/draft-guideline-34> | UK | National |
| <https://cicm.org.au/common/Uploaded%2520files/Assets/Professional%2520Documents/IC-10-Guidelines-for-Transport-of-Critically-Ill-Patients.pdf> | Australia | National |
| <https://www.ahajournals.org/doi/10.1161/CIR.0000000000001282> | USA | National |
| <https://www.phecit.ie/Custom/BSIDocumentSelector/Pages/DocumentViewer.aspx%3Fid%3DoGsVrspmiT0dOhDFFXZvIz0q5GYO7igwzB6buxHEgeD013qEh%25252bBwnpgYGEV7P6lBaIHN9i3gtl91GoBfn2HXa8jFQskt1vlTHQAXZzRWrqfnU%25252fs%25252fg%25252fPZHpree8grmUg3RC4N3AC5T6x%25252bc2B%25252bwT%25252fwQY8l62aTIOZl6Vd%25252fC6WUbYL9P5okOKJ%25252bPQ%25253d%25253d> | Ireland | National |
| <https://www.moh.gov.sa/en/Ministry/MediaCenter/Publications/Pages/IFCT_1.3.pdf> | Saudi Arabia | National |
| <https://emscimprovement.center/education-and-resources/interfacility-transfer/> | USA | National |
| <https://www.ahajournals.org/doi/10.1161/JAHA.122.026700> | USA | National |
| <https://executive.nhs.wales/networks-and-planning/critical-care/critical-care-docs/guidelines-for-the-transfer-of-the-critically-ill-adult/> | UK | National |
| <https://istitlaa.ncc.gov.sa/en/health/cbahi/NationalLaws/Pages/Article_014.aspx> | Saudi Arabia | National |
|  |  |  |
| USA | 9 |  |
| UK | 15 |  |
| Australia | 5 |  |
| South Africa | 0 |  |
| Ireland | 1 |  |
| Pacific | 0 |  |
| Saudi | 2 |  |
| Canada | 1 |  |
| **TOTAL** | **33** |  |
|  |  |  |
| National | 16 |  |
| Local | 18 |  |
| Literature | 11 |  |
| Insurance policy | 3 |  |
| Blog | 1 |  |
| Book | 1 |  |
| **TOTAL** | **50** |  |

Supplementary Material 3

# EXTRACCT Clinical Practice Guideline Development Process Introduction and Rationale

Clinical Process Guidelines (CPGs) are a pillar of the United States (US) Military’s Joint Trauma System (JTS). CPGs provide guidance to clinicians providing trauma and emergency care in the deployed environment around the management of various injury types/emergency medical conditions. CPGs serve numerous functions including: i) standardization of care and adherence to evidence-based care processes; ii) improvement in clinical performance/care quality; iii) longitudinal monitoring of patient outcomes including impact assessment associated with various interventions. Though deployed clinicians frequently provide care under austere conditions, the JTS CPGs are developed for casualties receiving care within a well-resourced military trauma system. The care of civilian casualties in conflict occurs within dramatically different set of resource constraints. Such differences include limited resource availability both with respect to equipment/materials and subspecialist expertise, limited critical care capabilities, limited organized modes of patient transport and lack of options for evacuation to higher echelons of care. With these differences in mind, the Explosive Weapons Trauma Care Collective (EXTRACCT) will develop a library of CPGs relevant to the care of civilian victims of blast injury in low-resource settings (LRS). These will be adapted from military CPGs in a manner that is appropriate to the available material and human resources of LRS.

# CPG Target Audience

Care for civilian victims of blast injury in LRS is provided by a range of medical personnel with varying levels of expertise in the management of complex trauma and varying levels of surgical training. EXTRACCT CPGs are designed for surgeons, medical doctors, clinical officers, and prehospital personnel working in LRS affected by explosive ordnance (EO) and other explosive weapons (EW) who may be required to care for blast injuries, particularly injury types outside of their standard scope of practice.

# CPG Topic Selection

The core EXTRACCT CPG topics were derived from the JTS CPGs that hold relevance to the management of blast injury in LRS. Future CPGs may be developed to address additional gaps in care. Pathways for proposal of new CPG topics include: requests from medical personnel providing care to EO/EW victims in LRS, requests from humanitarian practitioners and stakeholders within humanitarian mine action (HMA) or counter-IED (C-IED), or from EXTRACCT leadership and affiliates based on perceived need. CPG topic proposals will be reviewed by EXTRACCT’s clinical advisory board.

Criteria for CPG topic selection include the degree to which a proposed topic: i) addresses a perceived gap in knowledge or training; ii) reduces variability in care processes; iii) introduces evolution of best practices in trauma care to the target environment; iv) improves understanding of certain weaponry (e.g. incendiaries) and associated injury patterns.

Proposals for new CPG topics should be addressed to: [explosiveweaponstraumacare@gmail.com](mailto:explosiveweaponstraumacare@gmail.com)

# CPG Development Process

Approved CPG topics will proceed to development. A lead author and expert review committee will be identified. Experts within the committee will have relevant experience in the management of blast injury in resource-constrained environments and will be composed of equal representation from affected settings. A literature review will be conducted. Given the heterogeneity of individuals contributing to EXTRACCT CPG development, review methodology will be determined at the discretion of the lead author (i.e., rapid review and evidence synthesis versus formal scoping/systematic review). Review findings will be synthesized into a narrative summary and inform clinical guidance contained within the CPG.

# CPG Content Requirements

EXTRACCT CPGs will contain the following sections:

- Background
- Patient assessment
- Treatment guidelines
- Links to open-access materials on procedures relevant to clinical content within the CPG -References (source)
- References (additional clinical content/training material)

Given the broad variance in resource availability between settings affected by EO/EW, EXTRACCT CPGs will be formatted with matrices providing “good/better/best” guidance on various aspects of management. Guidance will be stratified in this manner based on available infrastructure and human/material resources.

# CPG Roles and Responsibilities

Each EXTRACCT CPG will be developed collaboratively with inputs from the following individuals whose responsibilities are enumerated below.

- Lead Author
  - Primary point of contact throughout CPG development
  - Communication with EXTRACCT leadership regarding selection of expert review panel (ERP)
  - Define CPG development timeline
  - Draft CPG outline and submit for initial review by EXTRACCT leadership •

Conduct literature review

- Draft CPG, submit to ERP for initial review
- Submit revised version of CPG based on ERP feedback to ERP and EXTRACCT Leadership
- CPG Expert Review Panel (ERP)
  - Review and revisions of initial CPG draft
- EXTRACCT Leadership/Clinical Advisory Board
  - Oversight of CPG development from concept to publication
  - Selection of CPG ERP
  - Liaise with lead author, review panel, and Journal of Conventional Weapons Destruction (JCWD) editorial team for publication
  - Dissemination of CPGs in EO/EW affected settings

# Core CPG Library

To generate an initial foundation of EXTRACCT CPGs relevant to the management of victims of EO/EW in low-resource environments, a core library will be developed. These topics have been selected from the JTS CPGs with applicability to the target environment. The core CPG library will be generated by trauma care specialists with appropriate expertise working in LRS on the following initial topics, with emphasis on prolonged field care:

- Airway management
- Hemorrhage control in the prehospital environment
- Damage control resuscitation
- Burn care
- Traumatic brain injury
- Thoracic trauma
- Abdominal trauma
- Pelvic trauma
- Genitourinary trauma
- Vascular injury
- Mangled extremity (to include decision-making around limb salvage)
  - Traumatic amputation
  - Compartment syndrome and fasciotomy
  - Extremity fracture management
  - Crush syndrome
- Embedded ordnance
- Embedded fragments
- Blast wound management
- Ocular trauma
- Acoustic blast trauma

# CPG Dissemination

EXTRACCT CPGs will be open access. Initial publication will occur in collaboration with the Journal of Conventional Weapons Destruction (JCWD). EXTRACCT leadership will collaborate with mine action operators and other HMA and health stakeholders to disseminate CPGs medical personnel caring for victims of EO/EW in LRS.

# CPG Updates

Existing CPGs will be reviewed routinely at five years or sooner if indicated on the basis of: i) evolution of trauma care best practices with applicability to the target environment; ii) needs identified by the target audience; iii) at discretion of CPG ERPs and EXTRACCT leadership.

Supplementary Material 4

*Authors’ experience and qualifications*

Dr Emma Butterfield is a doctor in intensive care and pre-hospital emergency medicine. She holds an MSc in trauma sciences, Diploma in Immediate Medical Care, Diploma in Retrieval and Transfer Medicine and Diploma in Tropical Medicine and Hygiene. She has worked extensively in aeromedical and road interfacility transfer in Australia and has published on risk assessment in aeromedical transfer of COVID patients. She is an examiner for the diploma in Retrieval and Transfer Medicine (RCSEd). She has co-ordinated and performed interfacility transfers in Sierra Leone and worked clinically in Mexico, Honduras and Iraq, and has previously published with EXTRACCT.

Dr Gavin Wooldridge is a doctor in pediatric intensive care medicine, with a range of retrieval experience across the UK, Australia and New Zealand. He has worked clinically, mostly for MSF, across Africa and the Middle East. A founding member of the Paediatric Blast Injury Partnership, with clinical experience working in conflict settings with a high burden of paediatric blast injury, and difficult interfacility transfers, he has previously collaborated with EXTRACCT in development of a Clinical Practice Guideline on Paediatric Blast Injury.

Dr Solomon Abraha Asfaw is a Senior Paramedic with Humanity & Inclusion (HI) Ethiopia, currently working under the Humanitarian Mine Action (HMA) project, where he provides advanced pre-hospital emergency care and medical support for field and EOD operations. He is actively involved in planning and operational delivery of emergency responses, patient stabilization, and medical evacuation in austere and high-risk environments. He is certified in Advanced Trauma Life Support (ATLS) with strong expertise in trauma assessment, airway management, and pre-hospital care for critically injured patients. His professional experience spans humanitarian emergencies, conflict-related trauma care, and emergency medical services, contributing practical frontline insight to guideline development and clinical decision-making in resource-limited settings.

Dr Mervat Al-Hilal is the Chief Medical Officer for the HALO Trust in Syria. She coordinates and trains staff and volunteers providing medical support to demining operations in Syria, including planning medical evacuations in an unstable post-conflict setting. She has ten years’ experience working for humanitarian NGOs delivering and co-ordinating clinical care and clinical training in Syria, including supervising and supporting teams of community health workers delivering care and transferring patients to higher levels of care during the civil war.

Dr Paul Reavley, Consultant in Paediatric Emergency Medicine. Bristol Royal Hospital for Children. Previously Consultant in Military Emergency Medicine and Rescue and Retrieval, Consultant in Pre-Hospital Emergency Care 2010-2018 UK, Defence Medical Services lead for deployed paediatric care 2010-2018. He is part of the Paediatric Blast Injury Partnership, a coalition between Save the Children UK, Imperial College London, World Innovation Summit for Health, Bristol Royal Hospital for Children, and kidconfident.org. The Partnership is dedicated to understanding battle-field injury in children, advocating for children in conflict, and supporting the clinicians in conflict zones who are treating child victims of war.

Professor Marius Rehn is a consultant anaesthesiologist working as an air ambulance doctor with the air ambulance at Oslo University Hospital, Norway, which performs both HEMS and secondary transfers, including neonatal critical care incubator transports. He conducts research on neonatal transport, physiology guided resuscitation and prehospital critical care. He is a professor in prehospital critical care at the University of Oslo and an adjunct professor at University of Southern Denmark. He has a background as a rescue man with the Royal Norwegian Air Force Search and Rescue helicopters and regularly provides medical direction and top cover for extreme remote environment teams (e.g. Patagonia, Antarctica, Greenland).

Dr Roman Shtybel is an Anesthesiologist, serving with the medical service of 12th Special Forces Brigade Azov. In this capacity, he contributed to the organization of damage control resuscitation as a part of the forward surgical group, critical care, and evacuation (including targeted evacuation). He has worked to enhance critical care delivery at the first point of medical contact, including driving the multimodal analgesia, infection control and advanced surgical care.

Dr Mardi Steere is a paediatric emergency physician and aeromedical retrieval specialist with more than 15 years of senior clinical, academic, and executive leadership experience across Australia, the United States, and East Africa. Since 2019, she has served as Executive General Manager, Medical and Retrieval Services for the Royal Flying Doctor Service South Australia/Northern Territory, with responsibility for all interhospital transports of adult, paediatric, and neonatal critical care patients across South Australia and Central Australia. She is a Fellow of the American College of Emergency Physicians, the American Academy of Pediatrics, and the Royal Australasian College of Physicians. Academic appointments include Associate Professor of Aeromedical Retrieval at Charles Darwin University, Assistant Professor of Emergency Medicine at the University of Florida, and Lecturer in Pediatric Emergency and Critical Care at the University of Nairobi, Kenya.

She has extensive experience developing interfacility transport and emergency care systems in resource-limited environments: she has worked as a consultant paediatric and neonatal retrieval physician with MedSTAR, South Australian Ambulance Service, and Clinical Director of a tertiary hospital in Kenya (2011-2019) and co-developed the Fellowship in Pediatric Emergency and Critical Care at the University of Nairobi/ Kijabe Hospital, launched in 2018, with critical care transport embedded as a core competency. She has published extensively on interfacility transport. She is the co-developer and inaugural faculty for Charles Darwin University’s Master and Graduate Certificate in Aeromedical Retrieval and is a board member of AMREF Flying Doctors Kenya since 2024.

A/Prof Benjamin W. Wachira is an emergency physician and academic with over 20 years of experience in emergency care delivery, education, research, and policy development. Since 2016 he is Assistant Professor and Program Director of Emergency Medicine at Aga Khan University, Nairobi, and Executive Director of the Emergency Medicine Kenya Foundation. He is an external examiner in Emergency Medicine qualifications in South Africa, Ghana and Rwanda, and led the development of the training program for the Diploma in Emergency Care in Kenya.

He has extensive experience in interfacility transport and emergency care systems development in resource-limited settings: he has worked with METRO Emergency Medical Services, the AMREF Flying Doctor Service, and the South African Red Cross Air Mercy Service. He has served on the Kenyan Emergency Medical Care Policy working group (from 2012) and the Kenyan National Emergency Medical Care Steering Committee (2021–2024). He was a lead contributor to the development of the Kenya Emergency Medical Care Policy and the Emergency Medical Care Strategy. He has authored several publications on emergency care referral pathways in resource limited settings and has presented internationally on the same.

Supplementary Material 5
